# Case Report: Microscope-assisted surgery of C7 nerve root cyst with neurological symptoms

**DOI:** 10.3389/fsurg.2022.977637

**Published:** 2022-08-04

**Authors:** Shenshen Hao, Hongke Li, Shuai Liu, Yating Guo, Honglei Chen, Zhifang Zhang, Shengli Dong

**Affiliations:** Department of Spine and Bone Oncology, General Hospital of Pingmei Shenma Medical Group, Pingdingshan, China

**Keywords:** cervical nerve root cyst, microscope-assisted surgery, treatment, neurological symptom, case report

## Abstract

**Background:**

Cervical nerve root cysts are rare and easily missed or misdiagnosed in clinical practice. Although conventional surgery is effective for cervical nerve root cysts, it is limited by the small surgical field of view and operating range, relatively difficult procedure, and incomplete cyst resection. Microscope-assisted magnification of the surgical field of vision reduces the difficulty and ensures complete cyst resection.

**Case presentation:**

A 58-year-old male patient was diagnosed with a cervical nerve root sleeve cyst on the right C7 vertebra with neurological symptoms. Microscope-assisted surgery was used as treatment. The cyst was completely removed by the microscope-assisted surgery, with satisfactory patient recovery. The surgery did not produce complications, such as spinal cord neurovascular injury, and the patient's symptoms disappeared quickly after surgery. During the 2-year follow-up, there was no complication and no recurrence.

**Conclusion:**

Microscope-assisted surgery in treatment of the C7 nerve root cyst could achieve a complete resection.

## Introduction

Cervical nerve root cysts, also known as meningeal or perineural cysts, are rare in clinical practice, especially causing discomfort in the neck, shoulder and upper limbs. The condition is relatively rare, with an incidence of only 1.5%–4.6%, and only 20% of patients are symptomatic (1). In the past, nerve root cysts were considered even more infrequent, which was actually due to an insufficient understanding of the disease or undeveloped examination methods (2). The basic feature of nerve root sheath cysts is that nerve fibers or ganglion cells are involved in the cyst wall and cavity (3). The etiology and pathogenesis of this disorder are still unclear. It is generally believed that abnormal arachnoid hyperplasia at the distal end of the spinal nerve root hinders the flow of normal cerebrospinal fluid, thereby triggering the formation of a cyst (4). Some specialists believe that traumatic hemorrhage in the nerve root sheath may lead to an expansion of the lacuna between the endoneurium and the cyst membrane, thereby inducing the formation of a cyst. This opinion hinges on the discovery of hemosiderin deposits in the cyst wall (5). Other specialists believe that the cyst may form due to inflammatory lesions of the nerve sheath caused by cervical disc herniation or degeneration. This opinion is based on the existence of nerve fiber tissue in the cyst wall (6). There is also a view that the cyst may form due to congenital diverticulum and elevated cerebrospinal fluid pressure (2).

We treated a case of C7 nerve root cyst with neurological symptoms by microscope-assisted surgery. The clinical symptoms of this patient were very similar to those of cervical spondylotic radiculopathy. If not carefully identified, this condition can easily be misdiagnosed, as had occurred with the patient on several occasions. Microscope-assisted magnification of the surgical field of vision reduces the difficulty of the operation and ensures complete resection of the cyst. We hope that this case report will serve as a reference for clinicians to diagnose and treat similar patients.

## Case presentation

**Figure 1:**
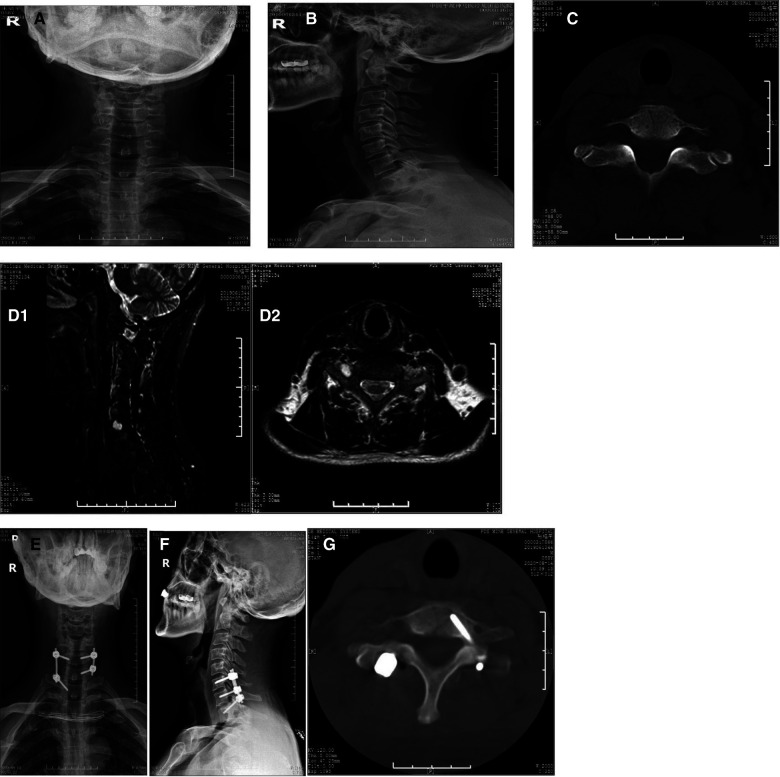
C7 nerve root cyst removed by microscope-assisted surgery. A 58-year-old man with a C7 nerve root cyst presented with neurological symptoms and was operated by microscope-assisted cystectomy with decompression of the cervical facet joints and internal fixation using screws. (**A,B**) Preoperative anterior (**A**) and lateral (**B**) Radiograph images of the cervical spine; no abnormality in the cervical spine is observed as shown on preoperative radiograph images. (**C**) Preoperative CT scans of the cervical spine. (**D**) Preoperative MRI scans of the cervical spine. The preoperative MRI shows abnormal signals in the right intervertebral foramina of the C6-7 segments, indicating a nerve root sleeve cyst. (**E,F**) Postoperative anterior (**E**) and lateral (**F**) radiograph images of the cervical spine; the postoperative radiograph images show that the cervical spine is well fixed. (**G**) The preoperative CT shows that the right neck of the C6-7 pedicle foramen is enlarged, the postoperative CT shows that the cervical spine is well fixed. R: right.

A 58-year-old man with a complaint of “neck and shoulder pain” was admitted to the hospital. The patient developed neck and shoulder pain and discomfort, with no obvious cause, about 1 year before. This was accompanied by pain and discomfort of the right upper limb, and the symptoms were relieved after rest. The neck pain began to worsen 3 months before admission, accompanied by a radiating pain in the right upper limb, which spread from the right shoulder to all fingers of the right hand, *via* the upper arm and the posterolateral forearm. The pain was paroxysmal and aggravated when the neck posture changed or when lying down. At the same time, the patient felt numbness of fingers 1–5 of the right hand; however, the following were absent: the feeling of walking on cotton wool, sense of tightness in the chest and abdomen, walking instability, headache and dizziness, hoarseness, difficulty in swallowing, fever, and night sweats. The patient received acupuncture, physiotherapy, and traction in a local hospital, but none of these treatments led to any obvious improvement. The physical examination revealed no evident deformity of the neck, but there was paraspinous cervical tenderness and limitations to the flexion and extension of the cervical spine, and to the abduction of the right shoulder. The patient developed hyperalgesia and hypoesthesia of the lateral upper arm and forearm of the right upper limb and fingers 1–5 of the right hand. In both Spurling's and Eaten's tests, there was a positive sign on the right side and a negative sign on the left side. There was no obvious muscle atrophy, low tension in both upper limbs, and negative Hoffmann's signs on either side. No abnormality in the cervical spine was observed as shown on the preoperative radiographs (Figures 1A, B). The preoperative computer tomography (CT) showed that the right neck of the C6–7 pedicle foramen was enlarged (Figure 1C). Magnetic resonance imaging (MRI) showed abnormal signals in the right intervertebral foramina of the C6–7 segments, indicating a nerve root sleeve cyst (Figure 1D). The patient was diagnosed with a cervical nerve root sleeve cyst of the right C7. After preoperative preparations, a surgical cystectomy with arthrectomy C6/7 right and screw internal fixation of the C6–7 was performed. The surgery proceeded smoothly without complications, such as spinal cord neurovascular injury, and the patient's symptoms disappeared quickly after surgery. The postoperative x-ray and CT scan images showed that the cervical spine was fixed well (Figures 1E–1G). The postoperative pathological results revealed a small amount of nerve fiber tissue in the cyst. There was no recurrence during the 2-year follow-up, and the right upper limb moved freely. Unfortunately, the patient was unwilling to undergo MRI re-examination; therefore, there were no more imaging data that could be presented.

## Discussion

The clinical symptoms of nerve root cysts are closely related to the degree of nerve root compression in the cyst. Smaller cysts have less tension, mostly leading to insignificant or mild clinical symptoms. Bigger cysts usually have higher cyst tension, more evident compression and lead to more serious symptoms (7), such as compression of the corresponding nerve roots and symptoms and signs associated with the ganglion. Clinical symptoms mainly include paroxysmal pain in the neck, shoulder and back, weakness and numbness of the upper limbs, and restricted movement, which are similar to those of radiculopathy, cervical disc herniation, and cervical spinal stenosis. In clinical practice, the symptoms of the neck and shoulder or upper limbs increase with changes in the neck posture or in the supine position (7). In this study, the patient presented with the specific clinical manifestations of nerve root sleeve cysts. Neck pain and right upper limb pain were paroxysmal, and pain was aggravated when changing the neck posture and lying down, which is related to changes in cerebrospinal fluid flow in these different positions.

MRI is currently recognized as the best imaging examination that can lead to a qualitative diagnosis prior to surgery in these conditions (8). On the MRI, nerve root cysts can appear as oval, long or dumbbell-shaped, occur on one or both sides of the nerve root sleeve and grow into the intervertebral foramen. The cysts show a uniform low signal on T1-weighted imaging and a uniform high signal on T2-weighted imaging. The MRI features are consistent with those of cerebrospinal fluid. In some cysts, nerve root shadows are thin and strip-shaped, which are better shown by cross-sectional T2-weighted imaging. Enhanced MRI scans can show unenhanced cysts and cyst fluids (9). Generally, x-ray or CT scans indicate no evident abnormalities associated with these cysts. The typical CT scan shows round cystic low-density shadows in the transition zone of the nerve roost or thickened nerve roots, and the corresponding intervertebral foramen or spinal canals can be enlarged.

An individualized treatment is required for nerve root sleeve cysts. Generally, asymptomatic patients who are accidentally discovered to have cysts do not need any treatment, and regular follow-up observation is enough. Surgical treatment is needed for symptomatic patients. Common surgical methods include laminectomy with decompression, electrocoagulation with cyst wall reinforcement, and percutaneous cyst fluid suction with injection of bioprotein glue into the cyst wall (10). In this study, we adopted microscope-assisted cystectomy with arthrectomy and screw internal fixation. The reasons for our choice of surgical approach are based on the following considerations. Firstly, the main location of the cyst is lateral to the pedicle, suggesting a condition in which only an arthrectomy can reveal the cyst. Therefore, the conventional method of laminectomy with decompression may result in a larger scope of surgical resection. Secondly, during routine surgery, the view of surgical field is easily limited. Because the field of vision observed from the surgeon's standing position is obviously blocked by the surrounding tissue or limited by the surgical incision. However, the lens of the microscope is closer to the cyst than the surgeon's eyes, which overcomes the difficulty of conventional visual field limitations. Thirdly, the removal of cysts requires certain conditions. With the aid of the microscope, the surgical field has been enlarged, which provides good conditions for direct cyst excision. In addition, we believe that direct cyst excision is an effective method that can directly relieve the etiology and reduce the risk of potential recurrence compared with the method of the electrocoagulation with cyst wall reinforcement. For the same consideration, we did not use the method of the percutaneous cyst fluid suction with injection of bioprotein glue into the cyst wall. Besides, the surgery inevitably destabilizes the cervical spine. The application of internal fixation can immediately stabilize the cervical spine, reduce the movement of the surgical site after surgery, reduce the risk of local nerve root irritation, and avoid the possible conditions that cause cysts, such as trauma and inflammation. Meanwhile, the rationality of the position of the screw should also be considered when placing the screw, so that the screw can stabilize the cervical spine without affecting the normal operation process. Therefore, these are the reasons for our choice of fixation.

## Conclusion

Cervical nerve root cysts, as in our case, are very rare disorders. Clinical manifestations of the disease are similar to those of common cervical spondylosis, which may easily lead to missed diagnosis and misdiagnosis. There are also some unique symptoms in this type of disorder: for example, pain of the neck and upper limb are aggravated or relieved with changes in the body position. When such symptoms appear clinically, cervical nerve root cysts should be considered. MRI can help confirm the diagnosis. Microscope-assisted surgery can help enlarge the surgical field, remove the cyst thoroughly, and reduce the area of exposure during surgery. The screw internal fixation helps restore the stability of the cervical spine immediately after the surgery. Unfortunately, only microscope-assisted surgery was conducted in this patient, and no further research on the etiology and pathogenesis of the disease was done. Therefore, future explorations are necessary.

## Data Availability

The original contributions presented in the study are included in the article/Supplementary Material, further inquiries can be directed to the corresponding author/s.
